# Submillimeter-Accurate Markerless Hand–Eye Calibration Based on a Robot’s Flange Features

**DOI:** 10.3390/s24041071

**Published:** 2024-02-07

**Authors:** Velibor Đalić, Vedran Jovanović, Petar Marić

**Affiliations:** Faculty of Electrical Engineering, University of Banja Luka, Patre 5, 78000 Banja Luka, Bosnia and Herzegovina; vedran.jovanovic@etf.unibl.org (V.J.); petar.maric@etf.unibl.org (P.M.)

**Keywords:** markerless hand–eye calibration, robot’s flange, TCP, point cloud, 3D scanner, 3D circle fitting

## Abstract

An accurate and reliable estimation of the transformation matrix between an optical sensor and a robot is a key aspect of the hand–eye system calibration process in vision-guided robotic applications. This paper presents a novel approach to markerless hand–eye calibration that achieves streamlined, flexible, and highly accurate results, even without error compensation. The calibration procedure is mainly based on using the robot’s tool center point (TCP) as the reference point. The TCP coordinate estimation is based on the robot’s flange point cloud, considering its geometrical features. A mathematical model streamlining the conventional marker-based hand–eye calibration is derived. Furthermore, a novel algorithm for the automatic estimation of the flange’s geometric features from its point cloud, based on a 3D circle fitting, the least square method, and a nearest neighbor (NN) approach, is proposed. The accuracy of the proposed algorithm is validated using a calibration setting ring as the ground truth. Furthermore, to establish the minimal required number and configuration of calibration points, the impact of the number and the selection of the unique robot’s flange positions on the calibration accuracy is investigated and validated by real-world experiments. Our experimental findings strongly indicate that our hand–eye system, employing the proposed algorithm, enables the estimation of the transformation between the robot and the 3D scanner with submillimeter accuracy, even when using the minimum of four non-coplanar points for calibration. Our approach improves the calibration accuracy by approximately four times compared to the state of the art, while eliminating the need for error compensation. Moreover, our calibration approach reduces the required number of the robot’s flange positions by approximately 40%, and even more if the calibration procedure utilizes just four properly selected flange positions. The presented findings introduce a more efficient hand–eye calibration procedure, offering a superior simplicity of implementation and increased precision in various robotic applications.

## 1. Introduction

Increasing the calculation accuracy of mathematical approaches and increasing the flexibility in usage by reducing the dependency on calibration objects have become major directions in development of hand–eye calibration algorithms recently [1]. However, achieving a high calibration accuracy is directly related to the careful design of the robot’s joint poses and the usage of specialized calibration objects with well-defined features [2,3], as well as robust mathematical models that can handle noise and outliers effectively [4,5]. On the other hand, increasing flexibility in practice is directly related to minimizing the dependency of the calibration methods on calibration objects or even eliminating the necessity for their usage at all. Another trend is the development of algorithms to automatically detect the need for recalibration, which can allow for continuous calibration adjustments based on detected changes in the environment [6,7].

Traditional calibration methods require placing physical markers or objects with predefined dimensions in the robot’s workspace [6,8,9,10]. These methods are proven to enable a highly accurate calibration, but various aspects, such as inaccuracies in the manufacturing of calibration markers, the non-planarity of calibration boards, the sensitivity of feature detection algorithms to ambient conditions, and the complexity and inaccuracy of algorithms needed for their detection and decoding, reduce their flexibility and efficiency [2]. To overcome the limitations imposed by utilizing the aforementioned standard calibration markers and objects, recently, there has been an increasing focus on developing hand–eye calibration methods that avoid the use of markers, known as markerless hand–eye calibration methods. Therefore, the main focus of this paper is to present a novel approach to markerless hand–eye calibration based on a robot’s flange geometrical features.

This paper is structured as follows. Section 2 provides an overview of markerless calibration methods and the proposed markerless calibration method objectives, prerequisites, and contributions. In Section 3, the problem statement for our calibration approach, followed by mathematical and notational conventions, is outlined. Relevant modifications, as well as the algorithm for processing the robot’s flange point cloud and extracting the coordinates of the robot’s TCP, are presented. Furthermore, the setup explanation is presented and the error metrics utilized in the verification process are introduced. The validation of the proposed algorithm, as well as the experimental results of the calibration, focusing on the strategic approach to flange positioning during the calibration process, is presented in Section 4. Section 5 includes a discussion of the results and comparison of our algorithm performances with others. Finally, Section 6 provides the paper’s concluding remarks.

## 2. Markerless Hand–Eye Calibration

In contrast to traditional marker-based calibration methods, markerless hand–eye calibration methods rely on the use of natural features and points present in the environment. The success of these calibration methods is underscored by their effective deployment in tasks such as robotic pick-and-place [11] and robotic surgery [12,13,14]. 

### 2.1. Markerless Calibration Methods

Recent advancements and methodologies have demonstrated steady progress in the field of markerless calibration. In a series of studies, Lambrecht [15,16] and Lee et al. [17] demonstrated the ability to estimate keypoints on a complete robot arm using RGB images. While Lambrecht used real-world data during training, Lee et al. relied on synthetic data. Further, they integrated the keypoint information with forward kinematics data to predict the robot’s pose accurately. In a related study, Zuo et al. [18] also proposed a keypoint-based detection network. However, their approach differed, as they directly regressed to the camera pose and unknown joint angles of a small, cost-effective manipulator through nonlinear optimization, without employing PnP (Perspective-n-Point) techniques. To train their network, they used synthetic data and addressed the reality gap by implementing domain adaptation techniques. Another noteworthy contribution by Labbe et al. [19] involved developing a method capable of predicting the robot pose and joint angles based on RGB images. Their approach employed an iterative CAD (Computer Added Design) to an image matching process, with a focus on utilizing a synthetic dataset for training purposes. Sefercik et al. [20] introduced a novel learning-based method for markerless extrinsic calibration, eliminating the need for simulation data and instead utilizing a depth camera. Their system only requires the end-effector to be visible within the visual system’s field of view, without the need for the majority of the robotic arm to be seen. Additionally, their method incorporates a 3D sensor, enabling the processing of depth data. In contrast, the other methods, which rely on 2D images, utilize the Perspective-n-Point algorithm to calculate affine transformations. By leveraging depth data, Sefercik et al.’s approach is grounded in real-world data, enabling them to apply the ICP (Iterative Closest Point) algorithm for refining position estimates, thereby reducing the necessity for synthetic or simulation data. In contrast, Valassakis et al. [21] presented a different approach using synthetic data and 2D images. They focused on a non-traditional robot–camera setup, deviating from the eye-to-hand configuration employed in the previously mentioned references. Their learning-based hand–eye calibration method involved performing hand-in-eye calibration via deep learning, directly regressing a camera pose from the images. However, the experiments conducted revealed that this approach lacked robustness in practical scenarios. Furthermore, a remarkable markerless and white-box method that ensures comprehensive positioning accuracy throughout the entire robot’s configuration space, named EasyHeC, is presented in [3]. To achieve this, the authors introduced two crucial technologies: differentiable rendering-based camera pose optimization and consistency-based joint space exploration. Through these innovations, they demonstrated significant progress in achieving precise end-to-end optimization of the calibration process, eliminating the need for the laborious manual design of robot joint poses. A hand–eye calibration method based on registration (RegHEC), eliminating the need for a precise calibration rig and allowing the use of arbitrary objects for eye-in-hand and eye-to-hand configuration, is presented in [22]. The authors presented an estimation of the hand–eye relation based on simultaneous aligning multiview point clouds of a common scene into the robot base frame. For achieving this goal, the authors proposed a novel variant of the ICP algorithm based on the Gauss–Newton method and Lie algebra, used multiview point clouds in the robot base frame for transforming, and refined the hand–eye relation through minimization of the Euclidian distance between corresponding points obtained by point-to-point estimation between point clouds. Markerless-based calibration has become very popular in video-based surgical navigation systems for estimating the spatial relation between the external tracking system and the optical axis of the surgical camera. A method utilizing point-to-line Procrustean registration for deriving the calibration data points is presented in [23]. Based on each frame of video tracked and the pivot-calibrated ball-tip stylus, the set of the 3D positions of the ball-tip (point) and its corresponding projection onto the video (line) is derived. Furthermore, to achieve high-quality hand–eye calibration, authors proposed a data sampling mechanism for optimization of the calibration fiducial spatial configuration. Moreover, a method for marker-less, intra-operative camera and hand–eye calibration is detailed in [14]. This innovative technique facilitates the calibration of both the camera and hand–eye coordination within a patient’s body without the need to remove the endoscope. The calibration processes are divided into pre-operative and intra-operative steps, eliminating the requirement for a calibration object. The hand–eye calibration is performed using the least square method in the pre-operative phase, and iteratively optimized utilizing the gradient descent algorithm in the intra-operative phase using the points from the previous estimation step with high visual errors. The estimated transformation is further used to render the surgical instrument-tip on the screen, allowing for immediate visual assessment of the transformation accuracy.

While these methods contribute to increasing the flexibility of calibration methods, their accuracy still remains below that of marker-based methods. However, the method proposed in [1] addresses the calibration problem through a two-step approach. Initially, it derives a precise closed-form solution focused on the translation equation, showcasing a superior accuracy and robustness compared to traditional methods. Subsequently, it reduces dependence on the calibration object to a single 3D-point using a similar translation-based formulation, minimizing the impact of estimation errors in the calibration object’s orientation while capitalizing on the increased accuracy and robustness achieved in the initial solution. The benefits of these proposals are recognized in both marker-based [24] and markerless methods [25], using a robot’s TCP as a single 3D point for calibration. To extract TCP coordinates, the authors in [24] proposed an algorithm based on the fitting of chessboard corner points, extracted using a sub-pixel corner extraction algorithm via iterative reweighted least square. On the other hand, a novel, direct, flange-based hand–eye calibration method, introduced in [25], involves the use of the standardized geometric features of the robot flange in both static and dynamic measurements, enabling direct hand–eye calibration and trajectory error tracking using the coordinates of the robot TCP. This approach is based on estimating the TCP coordinates from the flange point cloud using the known radius of the flange defined by the ISO standard [26], and the calibration accuracy is improved by utilizing the error compensation. 

### 2.2. Proposed Markerless Calibration Method 

To achieve simplified and submillimeter-accurate calibration, we proposed a novel approach to the calibration problem presented in [25]. We designed the hand–eye system and proposed a markerless calibration procedure. Compared to the methodology and results reported in [25], the main contributions of our study are related to increasing the calibration accuracy and streamlining the calibration procedure. Therefore, an error-compensation-free calibration procedure based on the least squares method, using a robot’s TCP as a calibration reference point, is proposed. The mathematical model of TCP-based calibration, simplifying the traditional maker-based hand–eye calibration model, is derived. To obtain the TCP coordinates, we propose a novel algorithm for the point cloud processing of the circular-shaped robot’s flange and an automatic estimation of its geometrical features, such as radius and center coordinates. The algorithm estimation method is based on the 3D circle fitting of the inner flange circle with an unknown radius using the NN algorithm, contrary to the method of the outer fitting circle using RANSAC with a known radius, reported in [25]. Furthermore, we proposed a novel accuracy validation procedure for the proposed algorithm using a calibration setting ring as the ground truth, exclusively due to its geometric similarity to the circular-shaped flanges of the robots, as well as the specified measurement accuracy. Finally, we conducted experiments on real-world data, and additionally investigated the influence of the number and choice of the robot’s unique flange positions on the calibration results. 

Our research is exclusively focused on the hand–eye calibration procedure, as a phase that precedes system utilization and is inevitable for achieving high precision in vision-guided applications. Considering that calibration, in general, is a process carried out according to a predefined protocol under predefined and controlled conditions, our procedure also entails several prerequisites that we deemed necessary for achieving the best possible results, as follows:

- Both the robot and the scanner must be calibrated according to the manufacturer’s recommendations before the hand–eye system calibration.
- The system is configured in an eye-to-hand setup, and the proposed procedure is designed to work only in static mode.
- The flange point cloud is obtained using the 3D scanner in accordance with the prescribed procedures outlined in the manual.
- The flange needs to be positioned in a way so that the upper surface of the flange is visible in the scanner’s field of view.

The main contributions of this paper are summarized as follows:

- An error-compensation-free hand–eye markerless calibration method based on a robot’s flange features,
- Achieving a high calibration accuracy using the proposed methodology,
- Reducing the complexity of the system calibration procedure and consistently providing submillimeter calibration accuracy, even when using just four properly selected flange positions for the calibration.

## 3. Method and Experimental Setup

The geometric features of the robot’s flange, defined by the ISO standard [26] and located at the end of the robot’s arm, show a potential to serve as a key points for high-precision hand–eye calibration without using markers. The usage of the robot’s flange as a hand–eye calibration reference object involves aligning the robot’s end-effector with a known reference point in the external environment. The choice of the reference point and its coordinate frame relative to the visual system is one of the crucial aspects for accurate calibration, particularly considering that the selection should rely on the identification of a fixed and easily recognizable point in the robot’s workspace that can serve as a reference point. When the tool is not attached to the robot’s end-effector, the TCP represents the center of the robot’s flange, as per the robot’s kinematic design [27]. Considering the geometry of the flange that is strictly defined by the ISO standardization [26] for each type of robot, in this study, the robot’s flange is used as the reference object, and its center as a reference point for the calibration. Therefore, the calibration procedure, mathematical model, and TCP estimation algorithm based on the flange’s point cloud processing are presented in the following sections, along with the experimental setup and validation procedures.

### 3.1. Problem Statement

**Figure 1:**
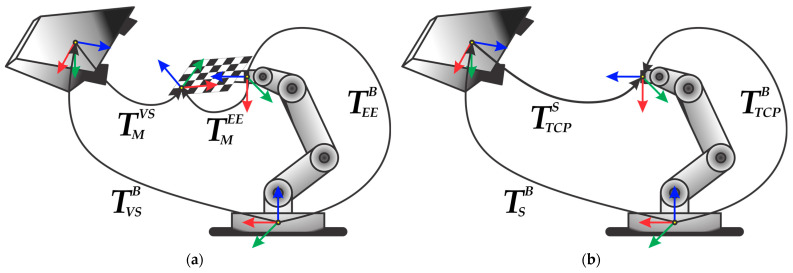
(**a**) The system suitable for traditional marker-based calibration; (**b**) the system employed for flange-based calibration without the tool attached to the robot’s end-effector.

Figure 1 illustrates the hand–eye systems, composed of an industrial robot and a visual sensor, comprising an eye-to-hand configuration.

Required transformations are defined for both systems, where B denotes the base of the robot, EE and TCP stand for the robot’s end-effector and tool center point, respectively, and M denotes the marker used for calibration, while VS and S represent the visual sensor and 3D scanner, respectively. For an easier understanding of the problem being solved, the mathematical and notational conventions, shown in Table 1, are followed.

All homogeneous transformations are 4 × 4 matrices, defined as:

(1) $$T_{n}^{m}=\left[\begin{array}{cc} R & t\\ 0^{T} & s \end{array}\right]$$

where the matrix R represents the 3 × 3 rotation matrix, t represents the three-element translation vector, and the scalar s represents the scaling factor.

This paper examines the calibration of the hand–eye system using the robot’s TCP, representing the flange upper plane center point, as a calibration reference point. It relies on a simplified mathematical model derived from the conventional marker-based hand-eye system, shown in Figure 1a, defined by:

(2) $$T_{EE}^{B}\cdot T_{M}^{EE}=T_{VS}^{B}\cdot T_{M}^{VS}$$

where the transformations $T_{VS}^{B}$ and $T_{M}^{EE}$ do not change throughout the calibration procedures and represent the unknown transformations that must be estimated, while the transformations $T_{EE}^{B}$ and $T_{M}^{VS}$ are previously calculated. Specifically, the transformation $T_{EE}^{B}$ is determined based on the kinematic parameters of the robot [27], whereas the estimation of the transformation $T_{M}^{VS}$ is performed through the previous visual sensor calibration procedure. 

As per Equation (2), the unidentified transformation $T_{VS}^{B}$ can be ascertained by examining a simultaneous set of two equations that are defined for two distinct positions of the calibration marker in the visual sensor’s field of view, designated as 1 and 2 in the subsequent equation:

(3) $${\left(T_{EE1}^{B}\right)}^{-1}\cdot T_{VS}^{B}\cdot T_{M1}^{VS}={\left(T_{EE2}^{B}\right)}^{-1}\cdot T_{VS}^{B}\cdot T_{M2}^{VS}$$

Furthermore, Equation (3) can be derived into a basic hand–eye calibration model:

(4) A·X=X·B

by assuming the following relations, $A=T_{EE2}^{B}\cdot {\left(T_{EE1}^{B}\right)}^{-1}$ $B=T_{M2}^{VS}\cdot {\left(T_{M1}^{VS}\right)}^{-1}$ and $X=T_{VS}^{B}$.

In contrast to a previously defined calibration model, the simplified calibration model presented in this paper is based on the estimation procedure of the transformation $T_{S}^{B}$ for the system depicted in Figure 1b, based on the direct estimation of the robot’s flange’s center coordinates, without the utilization of any additional calibration markers. Hence, the simplification of the marker-based to the flange-based system relies on the usage of the robot’s flange as a reference object. Considering that the flange-based hand–eye calibration requires a robot without a tool, it means that the coordinate systems of the robot’s end-effector (EE), marker (M), and TCP could be considered as being perfectly aligned with the origin in the TCP. Therefore, the transformation $T_{M}^{EE}$ defined for the system shown in Figure 1a transforms into an identity matrix $T_{M}^{EE}=I$ for the system shown in Figure 1b, while Equation (2) for a simplified flange-based calibration model, which relates the robot’s TCP to the corresponding visual observations obtained using the 3D scanner, can be written as follows:

(5) $$T_{TCP}^{S}={{(T}_{S}^{B})}^{-1}\cdot T_{TCP}^{B}$$

In Equation (5), the unknown transformation $T_{S}^{B}$ defines the relation between the robot and the scanner and is estimated during the hand–eye calibration procedure. In this paper, the proposed calibration process relies on point-set matching through a direct estimation of the TCP position without using its orientation. Therefore, to solve Equation (5), two independent sets of data points, $\left\{P_{ri}{=\left[x_{I},y_{I},z_{I},\;1\right]}^{T}\in R^{4}|i=1,2,\ldots ,m\right\}$ for the robot and $\left\{P_{si}={\left[x_{I},y_{I},z_{I},\;1\right]}^{T}\in R^{4}|i=1,2,\ldots ,m\right\}$ for the scanner, representing the translation vectors’ homogeneous coordinates of the transformations $T_{TCP}^{B}$ and $T_{TCP}^{S}$, respectively, need to be collected. The data points’ collection procedure is conducted as follows: the robot’s movements are controlled by issuing commands to the robot to move the flange to different spatial locations within the scanner’s field of view. Specifically, for each spatial location, the position of the robot’s flange is determined and its TCP point $\left\{P_{ri}\right\}$ is acquired. Simultaneously, the point cloud of the flange is acquired using the 3D scanner and the flange center coordinates $\left\{P_{si}\right\}$ are estimated using the proposed algorithm. These sets of data points are related by the following equation [28]:

(6) $$P_{ri}=R\cdot P_{si}+t+N_{i}$$

where R and t are the rotation matrix and translation vector of the resulting transformation matrix $T_{S}^{B}$, whereas vector $N_{i}$ represents the measuring noise. 

The unknown homogeneous transformation matrix $T_{S}^{B}$ is further evaluated by minimizing the least square problem as follows:

(7) $$e^{2}=\sum_{i=1}^{m}{\left\|P_{ri}-\left(R\cdot P_{si}+t\right)\right\|}^{2}$$

where the parameter m represents the cardinality of datasets $\left\{P_{ri}\right\}$ and $\left\{P_{si}\right\}$.

### 3.2. The Algorithm for Robot’s Flange TCP Estimation Based on Point Cloud Processing

**Figure 2:**
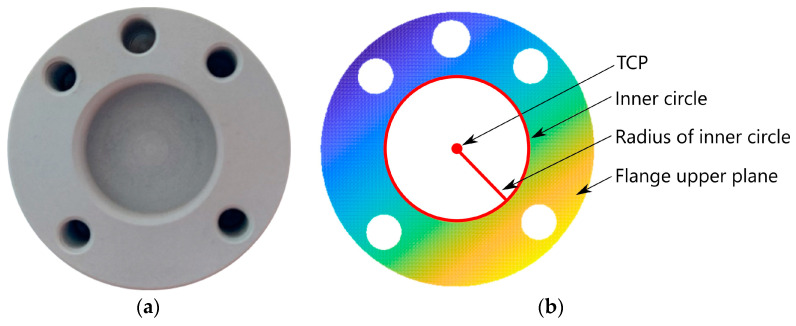
(**a**) The robot’s flange; (**b**) the robot’s flange point cloud.

The proposed algorithm focuses on the detection and estimation of the robot’s flange geometric features. The algorithm processes a point cloud of the robot’s flange, focusing on the flange upper plane points and providing an estimation of both the center coordinates (TCP) and the radius of the flange inner hole (circle), see Figure 2. 

**Figure 3:**
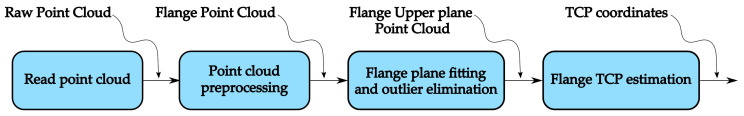
The block diagram of the proposed algorithm.

The block diagram depicting the main steps of the proposed algorithm is shown in Figure 3. 

Given that the flange is circular, the proposed algorithm utilizes the NN method to detect and extract points on the inner circle within the flange’s point cloud. The geometric characteristics of the flange are then estimated through a 3D circle fitting approach, employing SVD and the least square method, similar to the findings in [29].

**Figure 4:**
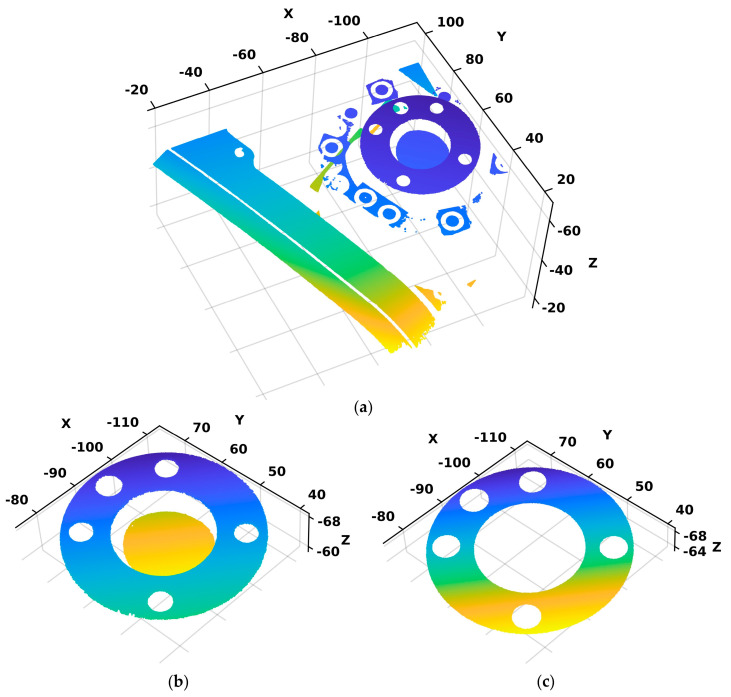
(**a**) Raw point cloud of the robot’s flange; (**b**) point cloud of the robot’s flange without outliers; and (**c**) point cloud obtained after the plane fitting process (upper plane of the flange).

The initial step of the algorithm entails the loading of the raw 3D point cloud of the flange acquired using a 3D scanner, depicted in Figure 4a. Then, to obtain a higher-quality point cloud with a focus on the flange, first, a pre-processing step is performed. Therefore, the outliers, representing unnecessary information from the working environment that is not of interest for calibration, are removed, see Figure 4b. Considering that the robot’s TCP coordinate system is located at the center of the inner circle of the flange’s upper plane, the subsequent processing of the point cloud involves estimation of the upper plane parameters using the MSAC (M-estimator Sample Consensus) algorithm [30]. Additionally, the algorithm extracts the points from the point cloud that belong to the upper plane, creating a new point cloud denoted by $CLP=\left\{{CLP}_{i}={\left[x_{i},y_{i},z_{i}\right]}^{T}\in R^{3}|i=1,2,\ldots ,n\right\}$, which enables a targeted analysis and calibration centered around this particular region, see Figure 4c.

The subsequent progression of the algorithm involves leveraging the obtained point cloud CLP as an input data for estimating the TCP of the flange. This process is implemented in three distinct phases. The first phase is intended to obtain an initial estimation of the flange’s inner circle TCP coordinates and the radius. The second phase aims to extract points from the point cloud located on the edge of the flange’s inner hole, as close as possible to the circle defined in the first phase. During the third phase, the optimal estimation of the flange TCP coordinates is provided by fitting a 3D circle through the points extracted during the second phase. A simplified illustration of each phase, focusing on points near the edge of the flange’s inner hole, as well as results obtained on a real point cloud and implementation details, are given as follows. 

**Phase 1:** The initial estimation of the flange’s TCP coordinates, denoted as $C_{f}$, is determined by averaging all the points in the point cloud CLP using the following equation:

(8) $$C_{f}=\left[\begin{array}{c} x_{f}\\ y_{f}\\ z_{f} \end{array}\right]=\frac{1}{n}\sum_{i=1}^{n}{CLP}_{i}$$

**Figure 5:**
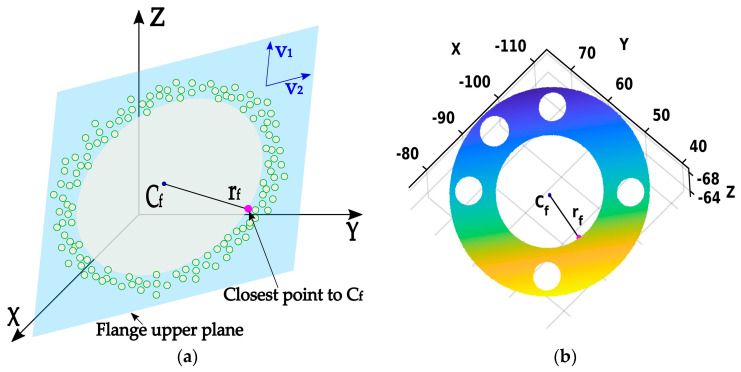
Phase 1—Initial estimation of the flange center coordinates $C_{f}$ (blue dot) and the radius of the inner circle $r_{f}$ (black line), and the point in the point cloud that is closest to initial center (pink dot). (**a**) Illustration of the Phase 1 result; (**b**) Result of the Phase 1 on the point cloud.

Furthermore, the NN method is used to find a point on the edge of the inner flange’s circle that is closest to the initially estimated center. The distance between the found point and the point $C_{f}$ is computed and used as the initial estimate for the radius of the inner circle, $r_{f}$. In Figure 5a, $C_{f}$ is shown as a blue dot, a pink dot is found to be the closest to the initially estimated center $C_{f}$, and the black line between the blue and pink dots represents an initial estimate for the radius $r_{f}$. The outcome of the first phase on a real point cloud is depicted in Figure 5b.

**Figure 6:**
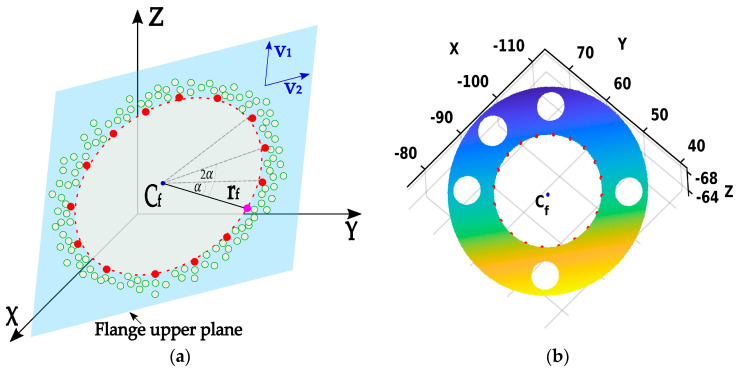
Phase 2—Points extraction (red border dots) closest to the circle defined with initial parameters. (**a**) Illustration of the Phase 2 result; (**b**) Result of the Phase 2 on the point cloud.

**Phase 2**: Once the initial parameters $C_{f}$ and $r_{f}$ are estimated, they are used to define a circle, shown with a red dashed line in Figure 6b. Furthermore, the NN method is used to extract the cloud points closest to the defined circle that lies on the inner edge of the flange, labeled as $CRP=\left\{{CRP}_{i}={\left[x_{i},y_{i},z_{i}\right]}^{T}\in R^{3}|i=1,2,\ldots ,n_{cp}\right\}$, where $n_{cp}$ represents the number of extracted points. The point extraction process is utilized, using linear sampling of the previously defined circle by the angle step α in range [0, 2π). The sampling angle is defined by $\alpha_{i}=i*\alpha =i*2\pi /n_{cp}$, resulting in evenly distributed discrete points on a circle across the interval [0, 2π). 

The extraction procedure of one point for a specific angle α can be explained, represented as follows:

(9) $${CRP}_{i}=NN(CLP,\;C_{f}+\sin\left(\alpha_{i}\right)\cdot r_{f}\cdot v_{1}+\cos\left(\alpha_{i}\right)\cdot r_{f}\cdot v_{2}).$$

The parameters $v_{1}$ and $v_{2}$ are the vectors that form an orthonormal basis of the flange’s upper plane, obtained using SVD on zero centered cloud points from CLP, as follows:

(10) $$USV^{T}=SVD(CLP-C_{f})$$

(11) $$v_{1}=V\left(:,1\right);v_{2}=V\left(:,2\right)$$

Figure 6b illustrates the second phase, where the red dots represent the extracted cloud points closest to the circle defined with parameters $C_{f}$, $r_{f}$, and α. The outcome of the second phase on a real point cloud is depicted in Figure 6b.

**Phase 3:** Finally, after selecting the inner points on the flange point cloud CRP, the next step of the algorithm involves obtaining a more accurate estimate of the flange’s center coordinates by fitting a circle through those points using the least square method. This procedure starts with projecting the mean zero centered points of CRP onto the new best-fitting plane based on the extracted points, using SVD, as follows:

(12) $$X_{p}=(CRP-C_{fnew})\cdot [v_{1new},v_{2new}]$$

where $C_{fnew}$, $v_{1new}$, and $v_{2new}$ are obtained utilizing Equations (8), (10) and (11) on the CRP cloud points. Subsequently, the optimal estimation of the center coordinates of a circle is obtained by fitting the circle with its center $\left(x_{c},y_{c}\right)$ and radius r to the 2D points obtained from Equation (12) in the following manner:

(13) $${\left(x_{i}-x_{c}\right)}^{2}+{\left(y_{i}-y_{c}\right)}^{2}=r^{2}$$

(14) $$p_{1}x_{i}+p_{2}y_{i}+p_{3}={x_{i}}^{2}+{y_{i}}^{2}$$

where $p_{1}=2x_{c}$, $p_{2}=2y_{c}$, and $p_{3}={{r^{2}-x}_{c}}^{2}-{y_{c}}^{2}$, while the coordinates of the projected points on the new plane are defined with ${x_{i}=X}_{p}\left(i,\;1\right);\;{y_{i}=X}_{p}\left(i,\;2\right);i=1,2,\ldots ,n_{cp}$. The vector of unknown parameters $p={\left[p_{1},p_{2},p_{3}\right]}^{T}$ can be further calculated by solving a system of linear equations given as:

(15) $$\left[\begin{array}{c} \begin{array}{ccc} x_{1} & y_{1} & 1 \end{array}\\ \begin{array}{ccc} x_{2} & y_{2} & 1 \end{array}\\ \vdots \\ \begin{array}{ccc} x_{n_{cp}} & y_{n_{cp}} & 1 \end{array} \end{array}\right]\cdot \left[\begin{array}{c} p_{1}\\ p_{2}\\ p_{3} \end{array}\right]=\left[\begin{array}{c} {x_{1}}^{2}+{y_{1}}^{2}\\ {x_{2}}^{2}+{y_{2}}^{2}\\ \vdots \\ {x_{n_{cp}}}^{2}+{y_{n_{cp}}}^{2} \end{array}\right]$$

The estimation of the optimal solution is performed by employing the least squares method, wherein the solutions are obtained in the form of:

(16) $$x_{c}=p_{1}/2;\;y_{c}=p_{2}/2$$

(17) $$r=\left(\sqrt{{p_{1}}^{2}+{p_{2}}^{2}+4p_{3}}\right)/2$$

Ultimately, the optimal estimation of the 3D TCP coordinates is determined by utilizing the ensuing equation:

(18) $${TCP}_{3D}=C_{fnew}+[v_{1new},v_{2new}]\cdot \left[\begin{array}{c} x_{c}\\ y_{c} \end{array}\right]$$

**Figure 7:**
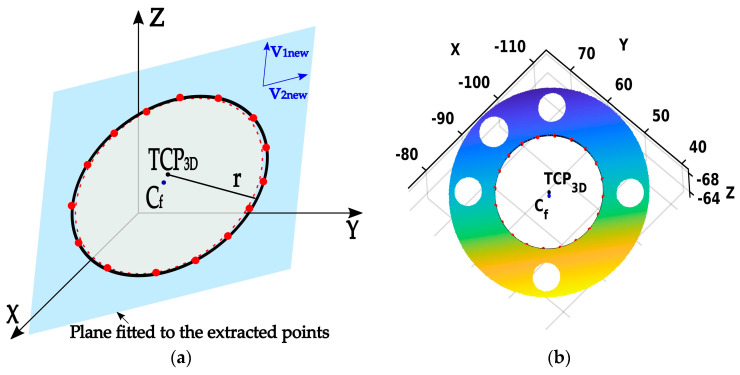
Phase 3—Optimal TCP estimation based on fitting a 3D circle through the extracted points. (**a**) Illustration of the Phase 3 result; (**b**) Result of the Phase 3 on the point cloud.

An illustration of the estimated TCP and radius is depicted in Figure 7a. Additionally, the circle fitted in the last phase of the algorithm compared to one determined with the initially estimated parameters, marked by a black solid and red dashed line, respectively, is emphasized. The outcome of the third phase on a real point cloud is depicted in Figure 7b.

### 3.3. Experimental Setup

**Figure 8:**
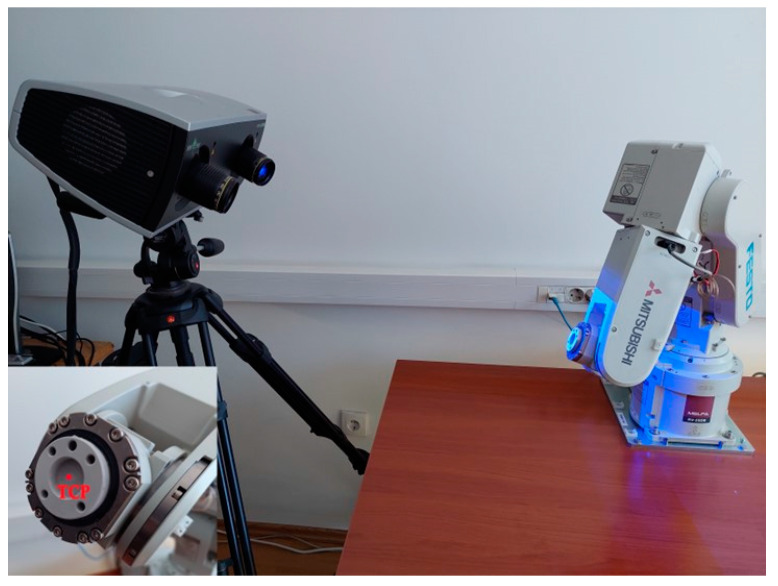
Photograph of the used experimental setup.

The experiments performed in this paper employ a hand–eye system that consists of a robotic arm and a structured light 3D optical scanner. Figure 8 depicts the complete experimental setup.

Considering the dimensions of the scanner and its working distance, as well as the necessity to obtain the point cloud of the robot’s flange directly, the hand–eye system is configured in an eye-to-hand configuration. This system configuration implies that the scanner is positioned stationary next to the robot and their mutual relation does not change. The eye-to-hand system configuration is the preferred approach in machine vision projects, providing the benefits of ease of installation, straightforward calculation, and a reduced likelihood of measurement errors [31]. Furthermore, this system is suitable for mobile and field use in situations in which it is not feasible or even possible to determine the position of the scanner relative to the robot in advance, as the flexibility required by use cases precludes attaching the scanner to the robot. In such scenarios, it is also often not feasible to perform complicated calibration procedures, as they increase the time and resource requirements in environments where the resources are constrained. Use cases where the system greatly benefits from the detachment of the scanner from the robot are applications such as robot-assisted surgical procedures [32,33,34], automatic robotic assembly [35], and object grasping [36]. 

The main characteristics of our system, including the robot and scanner, are highlighted as follows. 

The Mitsubishi RV2SDB robot arm by Mitsubishi Electric Corporation, Tokio, Japan, is used. The robot, with its six axes, has a reach of up to 504 mm and a repeatability of ±0.02 mm. To minimize the impact of system errors, and considering that the robot position error is mainly caused due to an error in zero position, the robot is pre-calibrated by positioning to the zero position and resetting the encoders’ values. The repeatability of the calibrated robot, as determined from conducted tests, measures 0.0214 mm, aligning with the manufacturer’s specifications. No consideration is given to any deviations in kinematic parameters or positioning errors at the end-effector of the robot, commanded by the means of a robotic controller.

The Comet LED 5M by Steinbichler Optotechnik GmbH, Neubeuern, Germany, which is based on blue LED structured-light technology, is utilized as a 3D scanner. The scanner is capable of capturing up to 5 million points within seconds, achieving a point cloud resolution of 0.02 mm. The scanner is based on a new and unique impulse scanning technique providing very high light output power, which improves the signal-to-noise ratio, obtaining remarkable measurement data and allowing instantaneous surface imaging. Moreover, it possesses the capability to employ various lenses, facilitating diverse field-of-view (FOV) configurations and quantifying volumes. The field-of-view named the FOV 250 configuration is selected, resulting in a measuring volume of 260 mm × 215 mm × 140 mm at a working distance of 760 mm. The scanner is calibrated and detailed information regarding the calibration procedure, accuracy assessment, and impact of the number of points detected during calibration on the system performance indices, as well as the scanner’s configuration and its optical characteristics, is presented in [37].

The algorithm is implemented using the Matlab programming environment exploiting the Computer Vision Toolbox, while the COMETplus 9.63 software, specifically designed to work seamlessly with the utilized scanner, is used for the scanning and preprocessing of the robot’s flange point cloud data purposes.

A scanning spray is used to improve the quality of the acquired point cloud. This prevents information loss that may occur due to reflections from the flange during the scanning process and maintains consistency in point cloud data acquisition.

### 3.4. Error Metrics 

The performances of the conducted experiments are measured as follows. 

The validation of the algorithm accuracy is quantified by the deviation between the estimated diameter, denoted as $d_{SRE}$, and the actual diameter of the setting ring, denoted as $d_{SRA}$, calculated using the following equation:

(19) $${error}_{SR}={\left\|d_{SRE}-d_{SRA}\right\|}_{2}$$

The calibration accuracy is determined by the deviations between the actual TCP position and the TCP position obtained using an estimated hand–eye calibration transformation matrix $T_{S}^{B}$, as follows: the center coordinates of the robot’s flanges, obtained using the proposed algorithm, are converted into coordinates relative to the robot by utilizing the estimated calibration transformation matrix $T_{S}^{B}$, as follows:

(20) $${P_{si}}^{\prime }=R\cdot P_{si}+t$$

where ${P_{si}}^{\prime }$ represents the estimated TCP position converted into coordinates relative to the robot, $P_{si}$ denotes the TCP coordinates relative to the scanner, and R and t are the rotation matrix and translation vector of the transformation matrix $T_{S}^{B}$ estimated through the hand–eye system calibration. Subsequently, the discrepancy between the estimated center coordinates of the flanges, converted into coordinates relative to the robot, and the actual robot’s TCP coordinates was used as an error model for the calibration accuracy analysis. This error model can be exemplified by the ensuing equation:

(21) $$error=\frac{1}{n_{fp}}\sum_{i=1}^{n_{fp}}\left|P_{ri}-\left(R\cdot P_{si}+t\right)\right|=\frac{1}{n_{fp}}\sum_{i=1}^{n_{fp}}\left|P_{ri}-{P_{si}}^{\prime }\right|$$

where error represents the averaged vector of discrepancies for each spatial coordinate, X, Y, and Z related to the robot coordinate system, and $n_{fp}$ is the number of flanges’ positions used in the validation.

## 4. Experimental Results

The devised and conducted experiments intended to assess the accuracy and efficacy of the proposed calibration approach are presented in this section. These experiments comprise (1) experimental setup preparation, (2) performance validation of the proposed algorithm using a setting ring, (3) calibration of the hand–eye system, (4) validation of the calibration results, (5) analysis of the number and the selection of the unique robot’s flange positions’ impact on the calibration outcomes, and (6) comparison of the obtained calibration results with the state of the art.

### 4.1. Algorithm Performance Validation Using a Setting Ring

**Figure 9:**
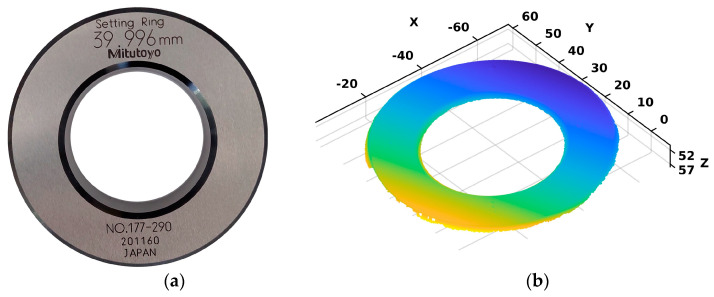
(**a**) The setting ring manufactured by Mitutoyo measures 40 mm in size and 15 mm in width. The setting ring’s outside diameter is 71 mm and it has an accuracy of 1.5 µm. The inner diameter of the setting ring, measuring 39.996 mm, is indicated on the ring. (**b**) The setting ring point cloud obtained using 3D scanner.

Considering that after a comprehensive search of the available literature, we could not identify an algorithm suitable as a benchmark for comparing the performance of the proposed algorithm, to evaluate the precision and repeatability of our proposed algorithm, we employed a setting ring as a reliable ground truth, as depicted in Figure 9a. The validation procedure entailed examining a 3D point cloud of the setting ring, which was acquired using the 3D scanner, as depicted in Figure 9b.

The setting ring chosen for validating the algorithm’s performance was primarily selected due to its close resemblance to the circular-shaped flanges of the robot. Considering that the fundamental goal of the algorithm is the precise estimation of the flange center coordinates, we deemed the setting ring as currently being the best solution for algorithm validation, both due to its geometric similarity and the reliability and accuracy it provides as ground truth, as it is, in fact, a calibration etalon for industrial metrology use.

Specifically, this setting ring serves as a precision-measuring tool for calibration and validation purposes, with a particular focus on the measurement of its inner diameter, which is precisely 39.996 mm. Therefore, we conducted experiments to verify the proposed algorithm’s accuracy in detecting and estimating both the center coordinates and the inner circle’s diameter of the ring. We performed these experiments across five different chosen positions and orientations of the setting ring, fulfilling the highlighted calibration procedure prerequisites, with each position undergoing 100 iterations. The selected number of positions for the setting ring covered a significant portion of the measurement volume within the scanner’s field of view. This sample size was utilized for subsequent validation experiments, as additional positions were deemed unlikely to contribute significantly to the validation process. It is very important to emphasize that the algorithm’s performance was not explicitly assessed in terms of the influence of additional effects, such as occlusion and light conditions, etc., as they are covered by the prerequisites listed in Section 2.

Furthermore, we conducted an analysis of the averaged outcomes to evaluate the precision and repeatability of the point cloud data loading and processing. The results are presented in Table 2, including the estimation of the center coordinates and diameter of the setting ring for each of the five positions.

**Figure 10:**
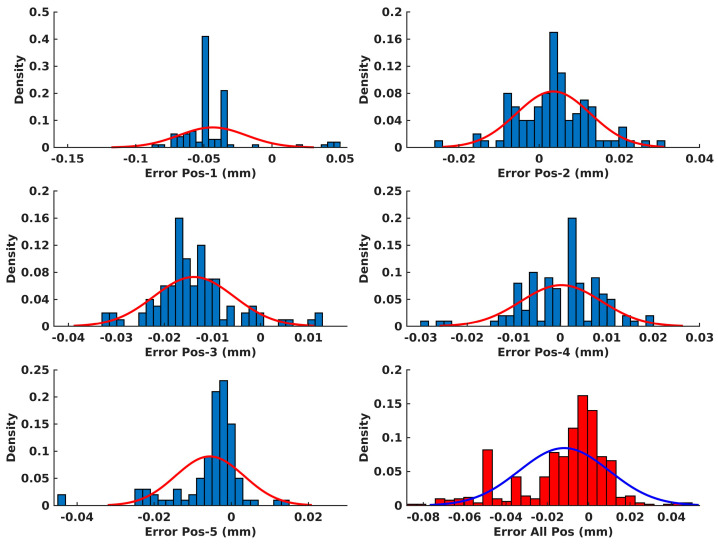
The error distributions of diameter measurements for each position of the setting ring.

Moreover, the error distributions of the diameter measurements for each position of the setting ring, as well as for all the positions together, are depicted in Figure 10. The error of the diameter measurements was obtained according to Equation (19), where $d_{SRE}$ represents the estimated setting ring diameter using the proposed algorithm and $d_{SRA}$ is actual value of the setting ring diameter, which is precisely 39.996 mm, according to the manufacturer specification.

### 4.2. Calibration Validation

The calibration experiments, utilizing the proposed hand–eye system and the proposed algorithm for the robot’s flange point cloud processing, were conducted in the following manner: initially, a set of 13 arbitrary positions for the robot flanges was defined within the common workspace, considering the robot’s space and the scanner’s field of view. Subsequently, the robot was positioned in each position and the point clouds of the robot’s flanges were obtained using the scanner. Each of these point clouds was processed by the proposed algorithm and the flange’s TCP coordinates were estimated related to the scanner. The processing and estimation were repeated 100 times for each position, and the mean values of the obtained results were further analyzed. 

Furthermore, the calibration of the hand–eye system was performed and a calibration matrix $T_{S}^{B}$, which defines the relation between the robot and the scanner, was estimated.

To investigate the impact of the number and the selection of the unique robot’s flange positions on the calibration outcomes, we conducted calibration experiments as follows. The calibration process was conducted for each combination of a unique set of k flange positions, $k\in \left\{4,5,6,7,8,9,10\right\}$. Next, the validation of the obtained calibration results was performed, according to the error model from Equation (21), for each calibration, using the estimated calibration matrix $T_{S}^{B}$ and the corresponding set points obtained from the remaining $n_{fp}=13-k$ flange positions which were not used for calibration. This process was based on determining the discrepancy between the estimated center coordinates using a proposed algorithm, converted into coordinates related to the robot and its actual TCP coordinates. 

**Figure 11:**
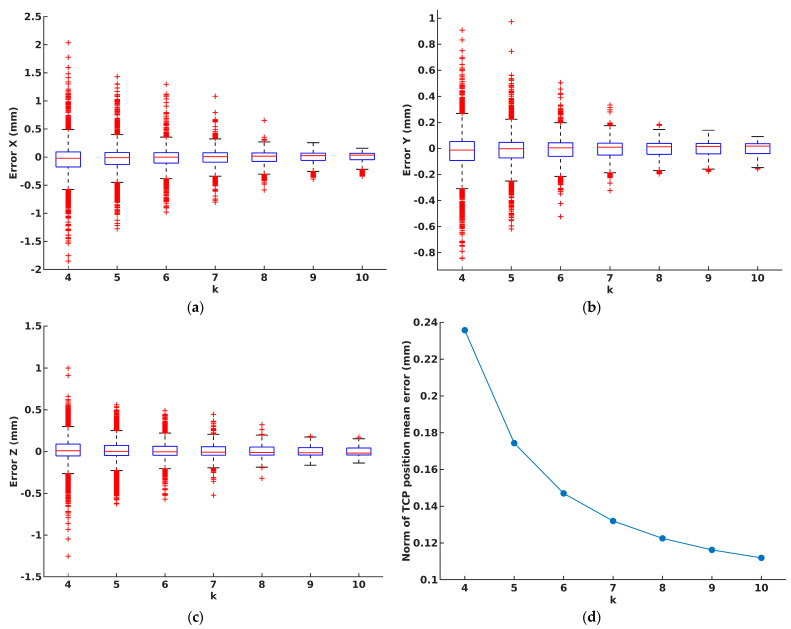
(**a**–**c**) The mean error between the estimated and actual TCP for each spatial coordinate X, Y, and Z, calculated for scenarios where from 4 to 10 flanges were used for calibration, and remaining flange positions for validation, respectively, (**d**) norm of TCP position mean error.

Throughout our experimentation, we conducted 130 scans of the robot flange using the scanner. Based on these scans, we conducted more than 7700 analyses of the system calibration results, varying the number of flange positions from 4 to 10, and validated the outcomes on positions that were not utilized in the calibration process. The validation results for each calibration experiment, representing the accuracy of the proposed calibration approach, are shown in Figure 11.

The norm of the presented error results for each scenario of calibration and corresponding validation is depicted in Figure 11d. 

**Figure 12:**
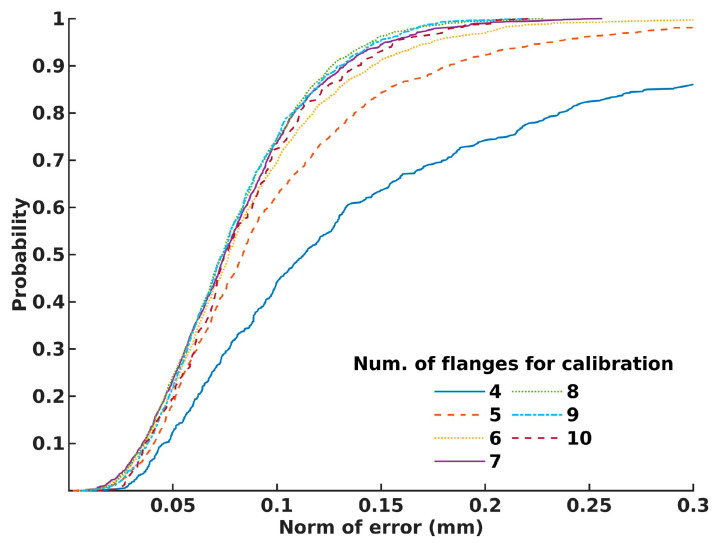
The probability for achieving accurate calibration, based on the number of flanges used in calibration process.

Considering the results and striving to derive general recommendations, Figure 12 illustrates the probability of attaining the desired calibration precision based on the number of flange positions used for calibration. 

## 5. Discussion

The study conducted in [25] emphasized the error compensation method to push calibration to achieve submillimeter accuracy. We designed a system and proposed the methodology of an error-compensation-free method for static markerless hand–eye system calibration, based on a robot’s flange features and achieving submillimeter accuracy. First, the mathematical model of the presented markerless hand–eye calibration was derived. Also, we proposed a novel algorithm for estimating TCP from the flange’s point cloud. Compared to the approach in [25], where the TCP coordinates were estimated based on fitting the flange’s outer circle with a known radius using RANSAC, we proposed the approach of identifying the inner circle in the flange point cloud and extracting the points lying on the identified circle using the NN method. Furthermore, our algorithm estimated the flange’s center coordinates by fitting a 3D circle using the least square method. This approach did not depend on the flange geometry and could be applied to any circular-shaped robot’s flange. 

Additionally, algorithm performance validation was quantified using the setting ring. The results from the algorithm performance validation demonstrated that the proposed algorithm provided highly accurate estimates of the inner circle diameter in all experiments, as shown in Table 2. Moreover, the greatest deviation from the mean diameter value was 0.025 mm. Since there is no way to verify the true values of the center coordinates of the setting ring in the coordinate system of the scanner, their absolute value was not crucial for validation. Therefore, the objective of the analysis was focused on assessing the repeatability of the algorithm. The findings, shown in Table 2, demonstrated a remarkable degree of repeatability, with the greatest deviation from the mean value being observed for a single coordinate of 0.013 mm. Furthermore, upon analyzing the error distribution for all the conducted setting ring positions, as depicted in Figure 10, it can be inferred that the proposed algorithm met expectations and yielded highly precise outcomes, both in terms of the repeatability of estimating the center coordinates and the estimation of the inner diameter of the setting ring.

Furthermore, calibration experiments were conducted and an analysis of the impact of the number of the robot’s flange positions and their mutual spatial configuration on the calibration outcomes was provided. Within this context, we embraced the established requirement that a minimum of four non-coplanar points is required for an estimation of the transformation between the robot and the scanner [25]. As expected, larger errors were noticed in cases where a smaller number of flanges was used for calibration, such as 4 or 5 flanges, as shown in Figure 11. On the other hand, the results of the remaining experiments, where the number of flanges used for calibration was larger than 5, showcased remarkable calibration precision, with errors remaining consistently below 0.2 mm per coordinate, regardless of the chosen arrangement of the flange positions. The norm error of the position discrepancy between the TCP position estimated by our algorithm and the actual TCP position remained consistent in all experiments. This consistency, as shown in Figure 11d, led to the conclusion that the proposed hand–eye calibration approach was suitable for achieving high-accuracy calibration of the proposed system, reducing the overall calibration procedure complexity.

Considering the previous studies which reported evaluations of the calibration methods without calibration markers with an error of about 2 mm [38,39], the best improvement in calibration results, to the authors’ best knowledge, was achieved in [25], with a reported error for static calibration method of about 1 mm in all three axes. The authors stated that a better accuracy could be achieved after error compensation. The comparison of our approach with the results achieved in [24,25] can be seen in Table 3.

Compared to the state of the art, the very important outcome of our approach is the one accomplished for the case that used just four flange’s positions for the system calibration, achieving submillimeter accuracy. According to the experimental results, our approach reduced the number of necessary flange positions and calibration datasets up to four times compared to the approach in [25], and almost three times compared to the approach in [24]. As the minimal number of non-coplanar points is four, this also represents the theoretically minimal number of points required for calibration in a 3D space, and the proposed system is fully usable with just four calibration positions. Also, it can be concluded that our approach contributes to reducing the complexity of the system and the calibration process, lowering the amount of time required for the system calibration procedure. Additionally, contrary to [25], our approach provides an error-compensation-free calibration with submillimeter accuracy. Finally, our results demonstrate a superior accuracy compared to the other two approaches, achieving submillimeter accuracy that was over five times better than that in [25], and over two times better compared to [24].

We have also proven that the probability of achieving submillimeter accuracy using our approach is over 95%, using a minimal four non-coplanar points for system calibration. Also, to achieve much stricter demands, we have shown that, to achieve an accuracy of 0.1 mm, the number of flange positions should be greater than six. By observing Figure 12, it is evident that selecting six or more flange positions provides calibration precision with a norm of error lower than 0.1 mm for over 75% of the chosen flange position combinations. Additionally, it is interesting to note that, even with a smaller number of flanges used for calibration, high precision can also be achieved, but in a lower percentage. This conclusion is expected, but also presents a challenge for operators, as they need to strategically choose positions that will generate calibration results with an acceptable error for their specific tasks. 

The method described in [25] also supports dynamic calibration, but the mean error obtained in that case was in the order of tens of mm, which is not favorable compared to other analyzed methods and imposes serious caveats on possible uses in the real-world. This method can utilize error compensation, which severely restricts the usage envelope of the method, but it still achieves the mean error of 0.15 mm, which is comparable to our proposed method without error compensation. As can be seen from previously presented results, our method can achieve an even higher accuracy by utilizing a lesser number of points than [25], even without error compensation.

Furthermore, it is important to emphasize that the quality of the calibration is influenced by the quality of the 3D scanned point cloud. Both the precision and accuracy of the scan directly influence the results of the calibration, as they are used as the ground truth, and any discrepancies or errors introduced in the scanning process will introduce unwanted errors in the calibration and affect the reliability of the system in use. It is, therefore, necessary to take precautions in the scanning process and consider both the specifications of the scanner and the scanning workflow and the best practices, as defined by the scanner manufacturer and the relevant scientific literature. To decrease errors introduced by scanning, we investigated the number of scanned flange positions and their locations in the working space of the scanner, as well as their influence on the results of the calibration process.

The main limitations of our approach compared to the state of the art include its restriction to static mode operation. Additionally, the inability to integrate CometLed effectively and robustly with the Matlab R2023a software environment requires additional effort in preparing the point clouds for further processing, such as outliers’ detection and removing procedures. These limitations may be considered in future work with a focus on improving the calibration performance both in the terms of accuracy and flexibility.

However, the results presented in this study serve as an excellent example that the strategy for choosing the number and the relative positioning of the flanges and the accurate and reliable processing of the flange point cloud are crucial aspects in the calibration process, especially if the main goal is to achieve a high precision and efficiency.

## 6. Conclusions

In this paper, we presented a static-mode error-compensation-free markerless hand–eye calibration method providing submillimeter accuracy. Our system was configured in eye-to-hand configuration and calibrated using the robot’s TCP estimated from the point cloud obtained by a 3D scanner. An algorithm for the estimation of the TCP coordinates, based on a 3D circle fitting using the least square and NN method, was proposed. The performance of the proposed algorithm was validated using the setting ring point cloud in a manner of diameter estimation and the repeatability of point cloud processing, demonstrating a high degree of accuracy, with the greatest deviation from the mean diameter value being 0.025 mm, as well as repeatability, with the greatest deviation from the mean value being observed for a single coordinate of 0.013 mm.

Furthermore, we proposed a calibration and validation strategy to optimize the process by reducing the number of required flange positions for achieving highly accurate calibration. Our experiments indicate that it is possible to achieve a submillimeter-accurate calibration of the hand–eye system without the need for error compensation, even when using just four properly chosen flange positions in the scanner’s field of view. To prove this, we conducted an analysis on the influence of the number of flanges and their mutual positions on the calibration precision. The obtained results demonstrated a high degree of accuracy, achieving mean errors below 0.2 mm per coordinate when using just four flange positions in the calibration process.

The presented findings have the potential for enhancement by considering the impact of robot precision on the system accuracy. Despite a robot’s good repeatability reducing positional variations in repetitive tasks, it does not eliminate the possibility of positional errors. While repeatability ensures consistent return to a designated position, it does not address systematic errors or inaccuracies. Therefore, even with outstanding robot repeatability, considering and, if necessary, addressing potential positional errors through additional robot calibration—comparing the actual TCP position with measurements from an independent calibration device—as well as employing other corrective measures, could result in improved calibration outcomes.

Compared to the state of the art, we improved the algorithm for circular-shaped flange point cloud processing, enabling the processing of the point cloud and the extraction of its geometric features without any knowledge of the flange geometry. In addition, we achieved a submillimeter calibration accuracy using the proposed algorithm, and improved the accuracy of previously reported results utilizing both marker-based and markerless approaches. We also simplified the calibration procedure, reducing the number of necessary flange positions while still providing submillimeter accuracy for properly chosen flange positions during the calibration procedure.

Finally, our approach shows great potential for significant improvements in the hand–eye calibration process, such as simplifying and streamlining the calibration procedure, reducing the preparation time, and minimizing the influence of human factors on the calibration process efficiency and accuracy.

## Figures and Tables

**Table 1:** Mathematical and notational conventions.

Example	Description
**Mathematical objects**	
s	Scalar values are shown by a non-boldface and lowercase font.
**r**	Vectors are shown in bold and lowercase. Unless otherwise indicated, all vectors are assumed to be column vectors.
$\left\|r\right\|$	The vector magnitudes are shown by double bars.
**R**	Matrices are indicated in bold and uppercase.
*P*	Points and positions are marked with capital italic letters.
$T_{n}^{m}$	The designation of homogeneous transformations is indicated by bold, italic, and uppercase letters, indicating the position of the frame *n* in relation to the frame *m*.
**Homogeneous Transformation**	
$T_{EE}^{B}$	The robot’s end effector related to the robot’s base.
$T_{M}^{EE}$	The calibration marker related to the robot’s end-effector.
$T_{VS}^{B}$	The visual sensor related to the robot’s base.
$T_{M}^{VS}$	The calibration marker related to the visual sensor.
$T_{TCP}^{B}$	The robot’s flange related to the robot’s base.
$T_{S}^{B}$	The 3D scanner related to the robot’s base.
$T_{TCP}^{S}$	The robot’s flange related to the 3D scanner.

**Table 2:** Algorithm validation on setting ring.

Ring Position	Diameter [mm]	X [mm]	Y [mm]	Z [mm]
1	39.95 ± 0.02	−36.21 ± 0.01	26.82 ± 0.01	55.03 ± 0.01
2	40.00 ± 0.01	−44.04 ± 0.01	27.42 ± 0.00	56.57 ± 0.01
3	39.98 ± 0.01	−52.67 ± 0.01	25.58 ± 0.00	59.71 ± 0.01
4	39.99 ± 0.01	−17.76 ± 0.00	18.63 ± 0.00	41.81 ± 0.01
5	39.99 ± 0.01	−0.39 ± 0.00	1.18 ± 0.00	6.29 ± 0.00

**Table 3:** Comparison with the state of the art.

Properties	Marker-Based [24]	Flange-Based [25]	Our Method
System configuration	Eye-to-hand	Eye-to-hand	Eye-to-hand
Vibration isolation table	No	Yes	No
Calibration mode	Static	Static and Dynamic	Static
Visual sensor	Stereo camera	3D scanner	3D scanner
Reference point	Robot flange TCP	Robot flange TCP	Robot flange TCP
Used robot TCP positions	80	16	From 4 to 10
Used num. of points pairs	10	16	From 4 to 10
TCP estimation	Multi-pixel 3D geometric centroid relocalization	Fitting outer circle of the flange with known radius	Fitting inner circle of the flange with unknown radius
Min. num. of points	10	16	4
Accuracy (Transl. err.)	0.45 mm	1 mm (static)	0.15 mm

## Data Availability

The data presented in this study are available on request from the corresponding author.

## References

[1] Grossmann B., Krger V. Continuous hand-eye calibration using 3D points. Proceedings of the 2017 IEEE 15th International Conference on Industrial Informatics (INDIN).

[2] Jiang J., Luo X., Luo Q., Qiao L., Li M. (2022). An overview of hand-eye calibration. Int. J. Adv. Manuf. Tech..

[3] Chen L., Qin Y., Zhou X., Su H. (2023). EasyHeC: Accurate and Automatic Hand-eye Calibration via Differentiable Rendering and Space Exploration. IEEE Robot. Autom. Lett..

[4] Sharifzadeh S., Biro I., Kinnell P. (2020). Robust hand-eye calibration of 2D laser sensors using a single-plane calibration artefact. Robot. Comput.-Integr. Manuf..

[5] Enebuse I., Foo M., Ibrahim B.S.K.K., Ahmed H., Supmak F., Eyobu O.S. (2021). A Comparative Review of Hand-Eye Calibration Techniques for Vision Guided Robots. IEEE Access.

[6] Lin W., Liang P., Luo G., Zhao Z., Zhang C. (2022). Research of Online Hand–Eye Calibration Method Based on ChArUco Board. Sensors.

[7] Pachtrachai K., Vasconcelos F., Edwards P., Stoyanov D. (2021). Learning to Calibrate—Estimating the Hand-eye Transformation Without Calibration Objects. IEEE Robot. Autom. Lett..

[8] Tsai R.Y., Lenz R.K. (1989). A new technique for fully autonomous and efficient 3D robotics hand/eye calibration. IEEE Trans. Robot. Autom..

[9] Daniilidis K., Bayro-Corrochano E. The dual quaternion approach to hand-eye calibration. Proceedings of the 13th International Conference on Pattern Recognition.

[10] Zhang Z., Zhang L., Yang G.-Z. (2017). A computationally efficient method for hand–eye calibration. Int. J. Comput. Assist. Radiol. Surg..

[11] Zhou Y., Fang Q., Zhao K., Tang D., Zhou H., Li G., Xiang X., Hu T. (2019). Robust Task-Oriented Markerless Extrinsic Calibration for Robotic Pick-and-Place Scenarios. IEEE Access.

[12] Shin S., Cho H., Yoon S., Park K., Kim Y., Park S., Kim L., Lee D. Markerless surgical robotic system for intracerebral hemorrhage surgery. Proceedings of the 2015 37th Annual International Conference of the IEEE Engineering in Medicine and Biology Society (EMBC).

[13] Hu J., Jones D., Valdastri P. Coordinate Calibration of a Dual-Arm Robot System by Visual Tool Tracking. Proceedings of the 2023 IEEE International Conference on Robotics and Automation (ICRA).

[14] Kalia M., Mathur P., Navab N., Salcudean S.E. (2019). Marker-less real-time intra-operative camera and hand-eye calibration procedure for surgical augmented reality. Healthc. Technol. Lett..

[15] Lambrecht J. Robust Few-Shot Pose Estimation of Articulated Robots using Monocular Cameras and Deep-Learning-based Keypoint Detection. Proceedings of the 2019 7th International Conference on Robot Intelligence Technology and Applications (RiTA).

[16] Lambrecht J., Kästner L. Towards the Usage of Synthetic Data for Marker-Less Pose Estimation of Articulated Robots in RGB Images. Proceedings of the 2019 19th International Conference on Advanced Robotics (ICAR).

[17] Lee T.E., Tremblay J., To T., Cheng J., Mosier T., Kroemer O., Fox D., Birchfield S. Camera-to-Robot Pose Estimation from a Single Image. Proceedings of the 2020 IEEE International Conference on Robotics and Automation (ICRA).

[18] Zuo Y., Qiu W., Xie L., Zhong F., Wang Y., Yuille A.L. CRAVES: Controlling Robotic Arm with a Vision-Based Economic System. Proceedings of the 2019 IEEE/CVF Conference on Computer Vision and Pattern Recognition (CVPR).

[19] Labbé Y., Carpentier J., Aubry M., Sivic J. Single-view robot pose and joint angle estimation via render & compare. Proceedings of the 2021 IEEE/CVF Conference on Computer Vision and Pattern Recognition (CVPR).

[20] Sefercik B.C., Akgun B. Learning Markerless Robot-Depth Camera Calibration and End-Effector Pose Estimation. Proceedings of the Conference on Robot Learning.

[21] Valassakis E., Dreczkowski K., Johns E. Learning eye-in-hand camera calibration from a single image. Proceedings of the Conference on Robot Learning.

[22] Xing S., Jing F., Tan M. (2023). Reconstruction-Based Hand–Eye Calibration Using Arbitrary Objects. IEEE Trans. Ind. Inform..

[23] Kemper T.N., Allen D.R., Rankin A., Peters T.M., Chen E.C. (2023). Open source video-based hand-eye calibration. Medical Imaging 2023: Image-Guided Procedures, Robotic Interventions, and Modeling.

[24] Fu J., Liu H., He M., Zhu D. (2022). A hand-eye calibration algorithm of binocular stereo vision based on multi-pixel 3D geometric centroid relocalization. J. Adv. Manuf. Sci. Technol..

[25] Wan F., Song C. (2020). Flange-Based Hand-Eye Calibration Using a 3D Camera With High Resolution, Accuracy, and Frame Rate. Front. Robot. AI.

[26] (2023). Manipulating Industrial Robots—Mechanical Interfaces—Part 1: Plates. https://www.iso.org/standard/36578.html.

[27] Denavit J., Hartenberg R.S. (1955). A kinematic notation for lower-pair mechanisms based on matrices. J. Appl. Mech..

[28] Arun K.S., Huang T.S., Blostein S.D. (1987). Least-Squares Fitting of Two 3-D Point Sets. IEEE Trans. Pattern Anal. Mach. Intell..

[29] Chernov N., Lesort C. (2005). Least Squares Fitting of Circles. J. Math. Imaging Vis..

[30] Torr P.H.S., Zisserman A. (2000). MLESAC: A New Robust Estimator with Application to Estimating Image Geometry. Comput. Vis. Image Underst..

[31] Su S., Gao S., Zhang D., Wang W. (2022). Research on the Hand–Eye Calibration Method of Variable Height and Analysis of Experimental Results Based on Rigid Transformation. Appl. Sci..

[32] Sun W., Liu J., Zhao Y., Zheng G. (2022). A Novel Point Set Registration-Based Hand–Eye Calibration Method for Robot-Assisted Surgery. Sensors.

[33] Liu J., Sun W., Zhao Y., Zheng G. (2022). Ultrasound Probe and Hand-Eye Calibrations for Robot-Assisted Needle Biopsy. Sensors.

[34] Qin Y., Geng P., Lv B., Meng Y., Song Z., Han J. (2022). Simultaneous Calibration of the Hand-Eye, Flange-Tool and Robot-Robot Relationship in Dual-Robot Collaboration Systems. Sensors.

[35] Liang P., Lin W., Luo G., Zhang C. (2022). Research of Hand–Eye System with 3D Vision towards Flexible Assembly Application. Electronics.

[36] Chen C.-S., Hu N.-T. (2023). Eye-in-Hand Robotic Arm Gripping System Based on Machine Learning and State Delay Optimization. Sensors.

[37] Jovanović V., Đalić V., Marić P. Accuracy Assessment of Structured-Light Based Industrial Optical Scanner. Proceedings of the 2022 21st International Symposium INFOTEH-JAHORINA (INFOTEH).

[38] Li W., Dong M., Lu N., Lou X., Sun P. (2018). Simultaneous Robot–World and Hand–Eye Calibration without a Calibration Object. Sensors.

[39] Wu L., Wang J., Qi L., Wu K., Ren H., Meng M.Q.H. (2016). Simultaneous Hand-Eye, Tool-Flange, and Robot-Robot Calibration for Comanipulation by Solving the AXB = YCZ Problem. IEEE Trans. Robot..

